# 气相色谱法测定中华绒螯蟹中脂肪酸组成与含量

**DOI:** 10.3724/SP.J.1123.2021.01032

**Published:** 2021-12-08

**Authors:** Zhaodong SHEN, Dongmei HUANG, Changling FANG, Hongli YE, Liangliang TIAN, Zi WU, Jun ZHANG

**Affiliations:** 1.上海海洋大学食品学院, 上海 201306; 1. College of Food Sciences and Technology, Shanghai Ocean University, Shanghai 201306, China; 2.中国水产科学研究院东海水产研究所, 农业部远洋与极地渔业创新重点实验室, 上海 200090; 2. Key Laboratory of Ocean and Polar Fisheries Innovation, Ministry of Agriculture, East China Sea Fishery Research Institute, Chinese Academy of Fishery Sciences, Shanghai 200090, China

**Keywords:** 气相色谱法, 脂肪酸, 中华绒螯蟹, gas chromatography (GC), fatty acids, Chinese mitten crabs

## Abstract

中华绒螯蟹中脂肪酸组成与含量的测定对评估其营养价值与品质具有重要意义,但面对种类繁多的脂肪酸提取试剂和甲酯化试剂,测定结果参差不齐,很难对中华绒螯蟹中丰富的脂肪酸准确定量。研究通过比较4种常见的脂肪提取试剂、2种脂肪酸甲酯化试剂,确定以氯仿-甲醇(1:1, v/v)为提取试剂,含2%硫酸的甲醇溶液为甲酯化试剂,建立了测定中华绒螯蟹肌肉中脂肪酸组成与含量的气相色谱分析方法。实验按照程序升温的条件,采用DM-2560毛细管色谱柱(100 m×0.25 mm×0.20 μm)分离37种脂肪酸,氢火焰离子化检测器(FID)检测,外标法定量。37种脂肪酸在0.5~100.0 μg/mL范围内线性关系良好,其相关系数(*R*^2^)为0.9981~0.9999,检出限(LOD)与定量限(LOQ)分别为0.01~0.02 mg/100 g和0.04~0.06 mg/100 g;以棕榈酸和硬脂酸进行加标回收验证,在1、2、10 mg/100 g 3个加标水平下的加标回收率为76.0%~97.5%,相对标准偏差(RSD, *n*=5)为3.31%~7.90%。该方法应用于中华绒螯蟹肌肉中脂肪酸组成与含量的测定,肌肉中共测得31种脂肪酸,碳链长度为12~24,脂肪酸总含量为281.03 mg/100 g,其中油酸、二十二碳六烯酸、二十碳五烯酸等为中华绒螯蟹肌肉中主要脂肪酸。该方法操作简便,试剂、样品用量少,且定性可靠,定量准确,能检测较多的脂肪酸种类,适用于中华绒螯蟹肌肉中脂肪酸组成与含量的快速检测。

## Abstract (en)

Determination of fatty acid compositions and contents in Chinese mitten crabs is of great significance to evaluate its nutritional value and quality. However, in the face of a wide range of fatty acid extraction and methyl esterification reagents, the measurement results are uneven, and it is difficult to accurately quantify the rich fatty acids in Chinese mitten crabs. In this paper, four kinds of oil extraction reagents and two kinds of methylating reagents, were investigated. Chloroform-methanol (1:1, v/v) was used as the extraction solvent, and methanol containing 2% sulfuric acid was used as the derivatization reagent. A method for the determination of fatty acids in the muscle of Chinese mitten crabs by gas chromatography was established. The experiment was carried out under the condition of programmed temperature rise, 37 kinds of fatty acids were separated on a DM-2560 capillary column (100 m×0.25 mm×0.20 μm), detected by hydrogen flame ionization detector (FID) and quantified by external standard method. The linear relationships of the 37 fatty acids were good in the range of 0.5-100.0 μg/mL. The correlation coefficients (*R*^2^) were 0.9981-0.9999. The limits of detection (LODs) and limits of quantification (LOQs) were 0.01-0.02 mg/100 g and 0.04-0.06 mg/100 g, respectively. The methodology was validated by palmitic acid and stearic acid. The recoveries were 76.0%-97.5%, and the relative standard deviations (RSD, *n*=5) were 3.31%-7.90% at the spiked levels of 1, 2 and 10 mg/100 g. The method was applied to the determination of fatty acid compositions and contents in the muscle of Chinese mitten crabs. A total of 31 kinds of fatty acids were detected. The length of carbon chain ranged from 12 to 24, and the total content of fatty acids reached 281.03 mg/100 g. Oleic acid, docosahexaenoic acid and eicosapentaenoic acid were the main fatty acids in the muscle of Chinese mitten crabs. Thus, this method provided accurate and reliable theoretical data for the determination of fatty acids in Chinese mitten crabs. This method has the advantages of simple operation, small amount of reagent and sample, reliable qualitative, accurate quantitative, detection of more fatty acid types. It is suitable for the rapid detection of fatty acid compositions and contents in muscle tissue of Chinese mitten crabs.

中华绒螯蟹又称大闸蟹、河蟹,是我国主要的淡水经济蟹类,广泛分布于中国沿海及通海的河流湖泊中,主要有辽河、黄河和长江三大水系。中华绒鳌蟹营养丰富,肉质鲜嫩,风味优良,尤其是以阳澄湖出产的大闸蟹最负盛名,是深受消费者喜爱的食品^[1]^,其蟹肉脂肪中富含不饱和脂肪酸(UFA),含量高达43%,其中二十碳五烯酸(EPA)和二十二碳六烯酸(DHA)为主要含量^[2]^。有研究表明,饱和脂肪酸(SFA)会引起血清总胆固醇升高,单不饱和脂肪酸(MUFA)却有保护心脏、降血糖、降低胆固醇、调节血脂和防止记忆下降等作用^[3]^,同时多不饱和脂肪酸(PUFA)有降血脂、防止动脉硬化、提高人体免疫机能等重要功能^[4]^。中华绒螯蟹中脂肪酸的组成与含量能够评估其营养价值与品质,因此建立一种高效、灵敏的脂肪酸含量测定分析方法尤为重要。

目前气相色谱法是分析脂肪酸组成应用最广的方法^[5,6]^,其具有分析时间短、分离效果好、检出限低、灵敏度高的特点^[7,8]^。脂肪酸提取^[9,10]^的常用溶液有氯仿-甲醇(TCM-MeOH)、二氯甲烷-甲醇(DCM-MeOH)、甲基叔丁基醚-甲醇(MTBE- MeOH)、乙醚-石油醚-甲醇溶液(DEE-PET-MeOH)等。常用衍生方法有酸催化^[11]^和碱催化^[12]^等。不同文献、不同实验室采取的脂肪酸提取和甲酯化试剂各不相同,测定结果参差不齐,很难实现脂肪酸的准确测定。目前,针对中华绒螯蟹中脂肪酸含量的检测方法较少,还需要进一步研究与开发。

因此,本文比较了4种常见的脂肪提取试剂及甲酯化试剂,确定了最合适的前处理方法,从而准确获得中华绒螯蟹的脂肪酸含量与组成特征。本方法的建立为中华绒螯蟹脂肪酸的测定提供了准确可靠的理论数据,是一种高效、灵敏、全面的分析方法。

## 1 实验部分

### 1.1 仪器与试剂

6890气相色谱仪,配有氢离子火焰检测器(FID)(美国Agilent公司); SG-8016A型数显水浴恒温振荡器(上海硕光电子科技有限公司); BT25S型十万分之一电子天平(赛多利斯科学仪器(北京)有限公司);数显型漩涡混合器(美国Tallboys公司)。

37种脂肪酸甲酯单标准溶液(100 mg/mL):丁酸甲酯(C4∶0)、己酸甲酯(C6∶0)、辛酸甲酯(C8∶0)、癸酸甲酯(C10∶0)、十一碳酸甲酯(C11∶0)、月桂酸甲酯(C12∶0)、十三碳酸甲酯(C13∶0)、豆蔻酸甲酯(C14∶0)、顺-9-十四碳烯酸甲酯(C14∶1)、十五碳酸甲酯(C15∶0)、顺-10-十五碳烯酸甲酯(C15∶1)、棕榈酸甲酯(C16∶0)、顺-9-十六碳烯酸甲酯(C16∶1)、十七碳酸甲酯(C17∶0)、顺-10-十七碳烯酸甲酯(C17∶1)、硬脂酸甲酯(C18∶0)、反-9-十八碳烯酸甲酯(C18∶1n9t)、油酸甲酯(C18∶1n9c)、反亚油酸甲酯(C18∶2n6t)、亚油酸甲酯(C18∶2n6c)、花生酸甲酯(C20∶0)、*γ*-亚麻酸甲酯(C18∶3n6)、顺-11-二十碳烯酸甲酯(C20∶1)、*α*-亚麻酸甲酯(C18∶3n3)、二十一烷酸甲酯(C21∶0)、顺-11,14二十碳二烯酸甲酯(C20∶2)、山嵛酸甲酯(C22∶0)、顺-8,11,14-二十碳三烯酸甲酯(C20∶3n6)、芥酸甲酯(C22∶1n9)、顺-11,4,17-二十碳三烯酸甲酯(C20∶3n3)、花生四烯酸甲酯(C20∶4n6)、二十三烷酸甲酯(C23∶0)、顺-13,16二十二碳二烯酸甲酯(C22∶2)、二十四烷酸甲酯(C24∶0)、二十碳五烯酸甲酯(C20∶5n3)、二十四碳烯酸甲酯(C24∶1)、二十二碳六烯酸甲酯(C22∶6n3)(纯度≥99%)和2种脂肪酸标准溶液(100 mg/mL):棕榈酸(C16∶0)、硬脂酸(C18∶0)(纯度≥99%)(美国NU-CHEK-PREP公司);甲醇、二氯甲烷、正己烷、正庚烷、甲基叔丁基醚(色谱纯,美国Merck公司);氯仿、硫酸、氨水、石油醚、乙醚(分析纯,美国Merck公司)。

### 1.2 标准溶液的配制

分别将100 mg/mL的37种脂肪酸甲酯单标准溶液用正庚烷配制成10 mg/mL的标准储备液,于-18 ℃棕色玻璃瓶中保存,有效期6个月;分别移取0.5 mL 37种脂肪酸甲酯单标准储备液,置于50 mL容量瓶中,用正庚烷配制成100 μg/mL的混合标准储备液,于-18 ℃棕色玻璃瓶中保存,有效期6个月;分别将100 mg/mL的棕榈酸、硬脂酸标准溶液用正庚烷配制成10 mg/mL的标准储备液,于-18 ℃棕色玻璃瓶中保存,有效期6个月。

将100 μg/mL的37种脂肪酸甲酯混合标准溶液,用正庚烷逐级稀释成浓度为0.1、0.2、0.5、1、5和10 μg/mL的系列混合标准工作液,于4 ℃棕色玻璃瓶中保存,有效期3个月。分别将10 mg/mL的棕榈酸、硬脂酸标准溶液用正庚烷配制成1 mg/mL的单标准工作液,于4 ℃棕色玻璃瓶中保存,有效期3个月。

### 1.3 实验方法

1.3.1 样品采集

中华绒螯蟹样品于阳澄湖养殖塘捕捞(东经120°45'19″,北纬31°25'25″),雌蟹和雄蟹各10只,平均重量为125.6 g,每个样品分别用自封袋封装编号并立即运回实验室。取其腿部及腹部肌肉混合均匀,将样品放入-18 ℃冰箱冷冻保存备用。

1.3.2 脂肪的提取

称取1 g中华绒螯蟹肌肉样品,置于玻璃管中,加入4 mL氯仿-甲醇(1∶1, v/v)、1 mL超纯水,涡旋90 s,于4 ℃以10000 r/min离心6 min,滴管吸取下层有机层溶液于玻璃管中,再向水层加入1 mL氯仿,重复提取一次,合并两次提取液,氮气吹干,得到干燥脂肪提取物。

1.3.3 脂肪酸甲酯化

向提取物中,加入2 mL 含2%硫酸的甲醇溶液,于70 ℃水浴加热50 min,完成后冷却至室温,再向样品溶液中加入2 mL蒸馏水,然后分2次加入2 mL正己烷,充分混合萃取,静置分层,取上层清液,再加入适量无水硫酸钠吸附水分,即得中华绒螯蟹肌肉中脂肪酸甲酯化溶液,待气相色谱分析。

1.3.4 气相色谱条件

DM-2560色谱柱(100 m×0.25 mm×0.20 μm); FID温度250 ℃;进样口温度250 ℃;载气:N_2_;分流比:10∶1;柱内流速:1.2 mL/min;色谱柱升温程序:初始温度120 ℃,保持1 min,以15 ℃/min升温至210 ℃,保持4 min,以5 ℃/min升温至240 ℃,保持10 min;进样量:1 μL。

## 2 结果与讨论

### 2.1 色谱柱的确定

中华绒螯蟹中的脂肪酸种类较多,构成复杂,本工作考察了填料为二氰丙基聚硅氧烷-非键合固定液的强极性毛细管色谱柱DM-2560 (100 m×0.25 mm×0.20 μm)与填料几乎等同于(5%苯基)-甲基聚硅氧烷的非极性苯基芳基聚合物的色谱柱DB-5MS (30 m×0.25 mm×0.20 μm)的分离效果。当采用DB-5MS毛细管色谱柱时,二十二碳烯酸、二十碳三烯酸与花生四烯酸甲酯不能分离,只出现一个峰;当采用DM-2560毛细管色谱柱时,37种脂肪酸甲酯在52 min内得到良好分离,且峰形良好(见图1),说明面对较多较复杂的脂肪酸时,选择长度较长、极性较强的DM-2560毛细管色谱柱才能达到良好的分离效果。因此实验采用DM-2560毛细管色谱柱进行分离。

**图1:**
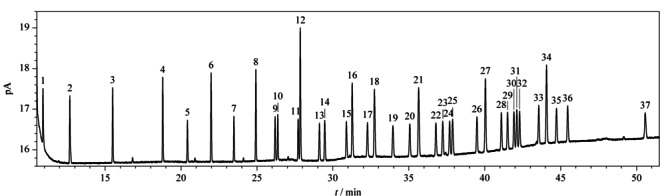
37种脂肪酸甲酯混合标准溶液(1 μg/mL)的色谱图

### 2.2 脂肪提取试剂的选择

面对种类繁多的提取试剂,本文将常见的脂肪提取试剂进行了比较,氯仿-甲醇溶液对脂蛋白、磷脂提取效率较高;二氯甲烷与氯仿性质相似但危险性较小;甲基叔丁基醚能与油脂能很好地混合;乙醚溶解脂肪能力强,应用较多,但乙醚能被2%的水饱和,含水乙醚提取脂肪的能力降低,并能使非脂成分溶解,从而被提取出来,使结果偏高,石油醚比乙醚溶解能力弱但允许含微量水分,两者混合使用效果更好。因此实验以中华绒螯蟹肌肉组织中含量较高的13种脂肪酸作为主要研究对象,通过不同的提取试剂比较这13种主要的脂肪酸的提取效果,来确定中华绒螯蟹脂肪酸的提取试剂,分别考察了氯仿-甲醇(1∶1, v/v)、二氯甲烷-甲醇(1∶1, v/v)、甲基叔丁基醚-甲醇(1∶1, v/v)、乙醚-石油醚-甲醇(1∶1∶2, v/v/v)对脂肪酸的提取效果。由图2可以看出,经氯仿-甲醇(1∶1, v/v)提取后,中华绒螯蟹中主要脂肪酸含量均明显高于其他3种试剂。因此本文采用氯仿-甲醇来提取中华绒螯蟹中的脂肪酸。

**图2:**
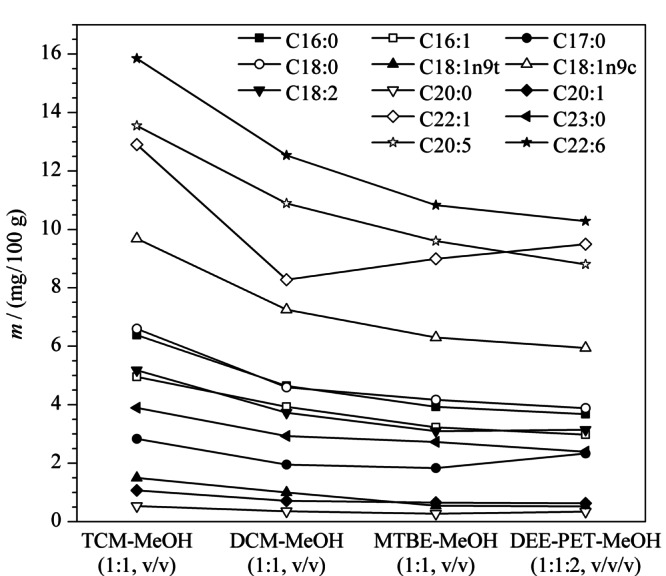
不同提取试剂对脂肪酸含量的影响

确定了氯仿-甲醇体系后,实验具体考察了氯仿与甲醇的体积比(1∶1、2∶1、1∶2)对提取效果的影响。从图3可以看出,氯仿-甲醇体积比为1∶1时提取的脂肪酸含量最高;氯仿含量过多,13种主要脂肪酸含量反而降低;氯仿含量过少,脂肪酸含量降低的更加明显。因此,确定氯仿-甲醇的体积比为1∶1。

**图3:**
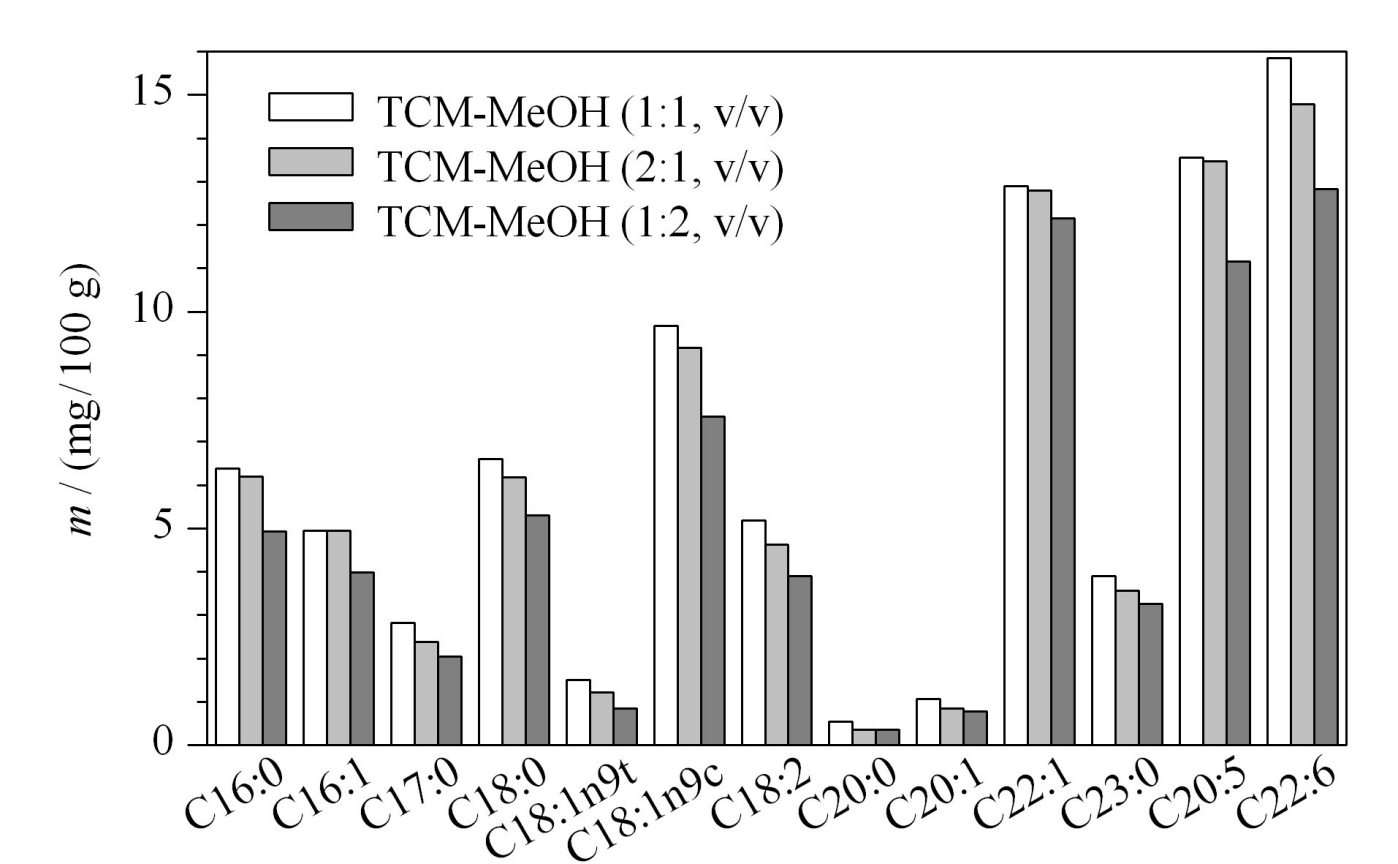
氯仿和甲醇的体积比对脂肪酸含量的影响

### 2.3 脂肪酸衍生化试剂的选择

在氯仿-甲醇溶液(1∶1, v/v)为提取试剂、70 ℃水浴的条件下,为研究不同甲酯化试剂的甲酯化效果,比较了含2%硫酸的甲醇溶液甲酯化50 min、含2%氢氧化钾的甲醇溶液甲酯化50 min以及酸碱结合甲酯化(先加入含2%硫酸的甲醇酯化25 min,再加入含2%氢氧化钾的甲醇酯化25 min)3种甲酯化方法对中华绒螯蟹干粉样品中脂肪酸含量的影响。从图4可以得出,中华绒螯蟹中的主要脂肪酸通过含硫酸的甲醇溶液甲酯化后含量显著高于其他两种甲酯化方法。

**图4:**
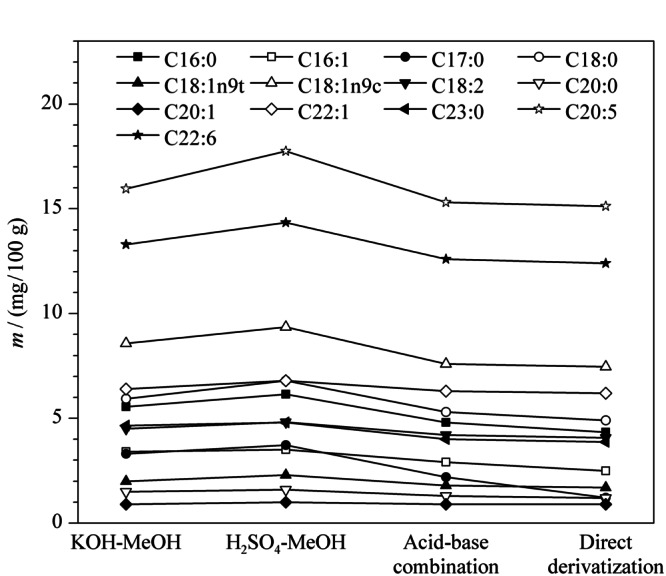
不同甲酯化方法对脂肪酸含量的影响

为探究先提取后甲酯化“两步”反应与直接甲酯化“一步”反应的甲酯化效果,同时对比了不经氯仿-甲醇(1∶1, v/v)提取,直接用含2%硫酸的甲醇甲酯化50 min的效果。从图4可以看出,“两步”反应虽然消耗更多的时间和试剂,但能更大程度提取并甲酯化中华绒螯蟹中的脂肪酸。因此本实验采用先提取后甲酯化的方法。

实验确定硫酸甲醇溶液为甲酯化试剂后,进一步探究含不同体积分数(1%、2%、5%、10%)的硫酸甲醇溶液的甲酯化效果。结果如图5所示,当硫酸体积分数为2%时主要脂肪酸含量达到最大值,此后增加硫酸的体积分数,脂肪酸含量反而降低。因此,本文最终确定采用含2%硫酸的甲醇溶液。

**图5:**
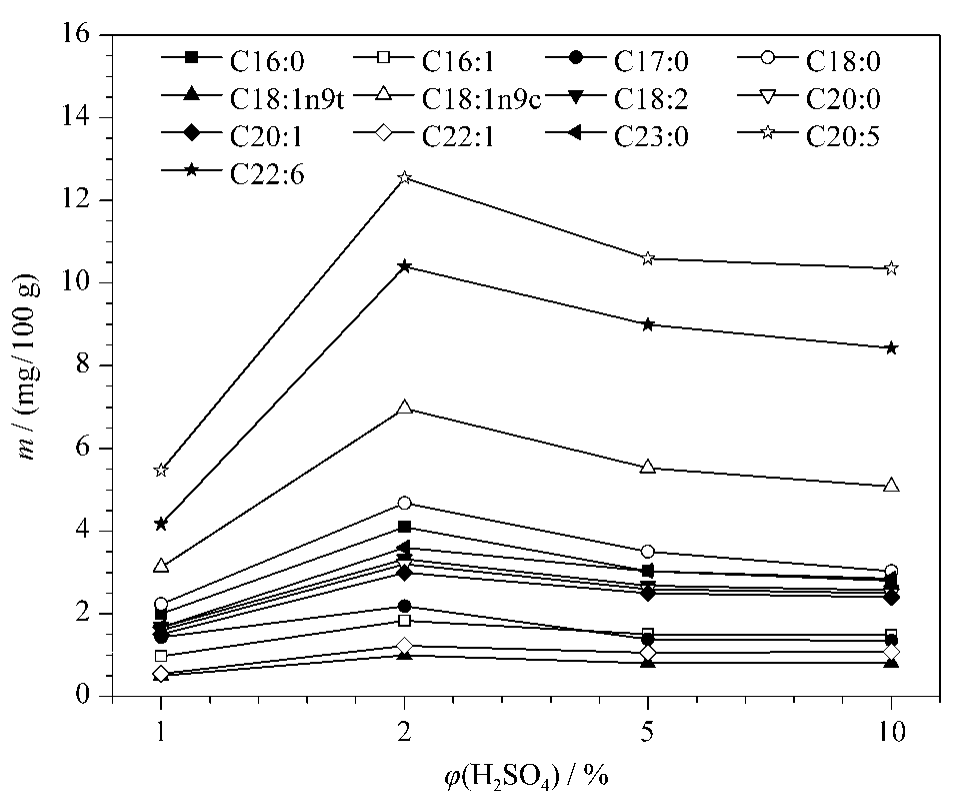
硫酸的体积分数对脂肪酸含量的影响

### 2.4 甲酯化水浴时间的优化

实验考察了不同水浴时间(30、40、50、60和70 min)对脂肪酸甲酯化的影响。结果如图6所示,中华绒螯蟹肌肉中的主要脂肪酸含量随着水浴时间的增加而增加,最终在50 min时达到最大值,此后增加水浴时间,脂肪酸含量反而降低。因此本文优化后的甲酯化的水浴时间为50 min。

**图6:**
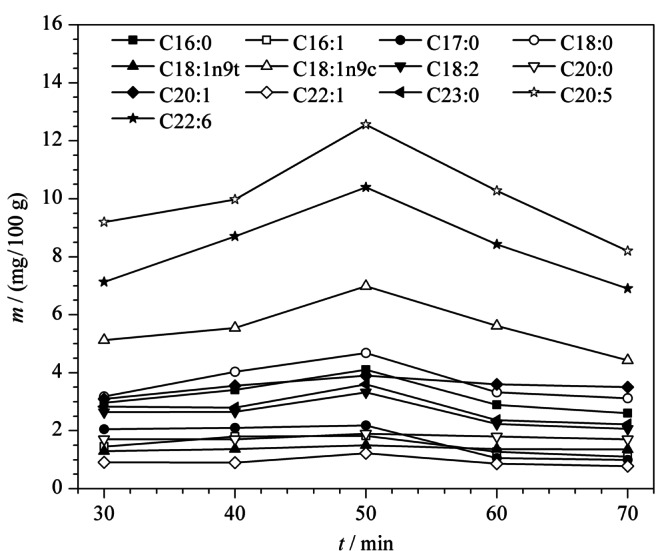
不同水浴时间对脂肪酸含量的影响

### 2.5 方法学考察

2.5.1 线性范围、检出限及定量限

对37种脂肪酸甲酯混合标准溶液进行测定,以脂肪酸甲酯的质量浓度(*x*, μg/mL)为横坐标,峰面积(*y*)为纵坐标绘制标准曲线(见表1)。结果表明,脂肪酸甲酯在0.5~100 μg/mL线性范围内线性相关系数(*R*^2^)为0.9981~0.9999。

**表1:** 37种脂肪酸甲酯的回归方程、相关系数、检出限和定量限

No.	Compound	Regression equation	R^2^	LOD/ (mg/100 g)	LOQ/ (mg/100 g)
1	C4∶0	y=2.127x+0.385	0.9982	0.01	0.04
2	C6∶0	y=1.418x+0.043	0.9997	0.01	0.04
3	C8∶0	y=1.406x+0.062	0.9994	0.01	0.04
4	C10∶0	y=1.388x+0.145	0.9998	0.01	0.04
5	C11∶0	y=0.681x+0.146	0.9992	0.02	0.06
6	C12∶0	y=1.430x+0.127	0.9993	0.01	0.04
7	C13∶0	y=0.711x+0.131	0.9996	0.02	0.06
8	C14∶0	y=1.446x+0.172	0.9999	0.01	0.04
9	C14∶1	y=0.711x+0.134	0.9999	0.02	0.06
10	C15∶0	y=0.739x+0.086	0.9987	0.02	0.06
11	C15∶1	y=0.727x+0.118	0.9999	0.02	0.06
12	C16∶0	y=2.244x+0.251	0.9995	0.01	0.04
13	C16∶1	y=0.714x+0.102	0.9988	0.02	0.06
14	C17∶0	y=0.767x+0.066	0.9999	0.02	0.06
15	C17∶1	y=0.751x+0.097	0.9989	0.02	0.06
16	C18∶0	y=1.501x+0.143	0.9989	0.01	0.04
17	C18∶1n9t	y=0.751x+0.064	0.9983	0.02	0.06
18	C18∶1n9c	y=1.536x+0.161	0.9981	0.01	0.04
19	C18∶2n6t	y=0.746x+0.100	0.9999	0.02	0.06
20	C18∶2n6c	y=0.773x+0.101	0.9986	0.02	0.06
21	C20∶0	y=1.528x+0.152	0.9987	0.01	0.04
22	C18∶3n6	y=0.742x+0.173	0.9997	0.02	0.06
23	C20∶1	y=0.781x+0.145	0.9996	0.02	0.06
24	C18∶3n3	y=0.784x+0.139	0.9997	0.02	0.06
25	C21∶0	y=0.762x+0.136	0.9996	0.02	0.06
26	C20∶2	y=0.762x+0.130	0.9999	0.02	0.06
27	C22∶0	y=1.578x+0.171	0.9987	0.01	0.04
28	C20∶3n6	y=0.769x+0.139	0.9989	0.02	0.06
29	C22∶1n9	y=0.762x+0.158	0.9992	0.02	0.06
30	C20∶3n3	y=1.568x+0.126	0.9993	0.02	0.06
31	C20∶4n6	y=0.746x+0.113	0.9998	0.02	0.06
32	C23∶0	y=0.784x+0.104	0.9999	0.02	0.06
33	C22∶2	y=0.784x+0.101	0.9999	0.02	0.06
34	C24∶0	y=0.776x+0.158	0.9989	0.01	0.04
35	C20∶5n3	y=1.648x+0.171	0.9991	0.02	0.50
36	C24∶1	y=0.772x+0.152	0.9998	0.02	0.06
37	C22∶6n3	y=0.673x+0.126	0.9999	0.02	0.06

*y*: peak area; *x*: mass concentration, μg/mL.

分别对37种脂肪酸甲酯标准溶液逐级稀释,进样测定,分别以3倍和10倍信噪比时的含量为检出限(LOD)和定量限(LOQ)。结果显示,37种脂肪酸甲酯的LOD和LOQ分别为0.01~0.02 mg/100 g和0.04~0.06 mg/100 g(见表1)。

2.5.2 加标回收率和精密度

中华绒螯蟹中脂肪酸种类丰富,实验选取了含量较高的棕榈酸和硬脂酸,进行了加标回收率试验。称取1 g空白中华绒螯蟹肌肉样品,在1、2、10 mg/100 g 3个加标水平下加入棕榈酸、硬脂酸两种脂肪酸标准溶液,每个水平平行5次。结果发现,这两种脂肪酸平均加样回收率为76%~97.5%, RSD为3.31%~7.90%(见表2),表明方法准确度高,精密度良好。

**表2:** 在3个加标水平下棕榈酸、硬脂酸的回收率及相对标准偏差(*n*=5)

Compound	Background/ (mg/100 g)	Added/ (mg/100 g)	Found/ (mg/100 g)	Recovery/ %	RSD/ %
C16∶0	16.19	1	16.95	76.0	6.80
		2	18.14	97.5	7.90
		10	25.04	88.5	3.31
C18∶0	10.02	1	10.95	93.0	5.78
		2	11.93	95.5	6.15
		10	19.03	90.1	3.55

### 2.6 实际样品检测

准确称取1 g中华绒螯蟹肌肉样品5份,测定其中各脂肪酸含量(见表3)。结果表明,共检出31种脂肪酸,碳链长度为12~24,脂肪酸总含量达到281.03 mg/100 g, DHA、EPA、油酸、亚油酸、棕榈酸等是中华绒螯蟹肌肉中的主要脂肪酸。其中饱和脂肪酸占总脂肪酸的17.01%,单不饱和脂肪酸为24.72%,多不饱和脂肪酸为58.27%,中华绒螯蟹中脂肪酸的营养价值主要指标为EPA和DHA,含量高达43.89%。各类脂肪酸特征和Jiang等^[13]^和王潇等^[14]^结果相似,其中多不饱和脂肪酸*ω*-3与*ω*-6(不饱和脂肪酸中第一个双键的位置命名的一系列脂肪酸)的比值为4.74,远高于国际粮农组织(FAO)和世界卫生组织(WHO)推荐的*ω*-3/*ω*-6日常膳食比(0.1~0.2)^[15]^,较高的*ω*-3/*ω*-6能有效降低血脂,抑制血小板凝集,降低心血管疾病的发病率^[16]^。综上所述,中华绒螯蟹中脂肪酸种类丰富,脂肪酸不饱和度高,具有降血脂、软化血管、抑制冠心病和血栓形成等功能,是优质的食用资源。

**表3:** 中华绒螯蟹肌肉中脂肪酸含量与百分比(*n*=5)

Compound	Content/ (mg/100 g)	Percentage composition/%	RSD/%
C12∶0	0.12	0.04	2.70
C14∶0	1.60	0.57	3.11
C14∶1	0.53	0.19	3.96
C15∶0	1.20	0.43	2.68
C15∶1	0.09	0.03	2.08
C16∶0	16.19	5.81	4.36
C16∶1	19.92	7.07	5.85
C17∶0	5.75	2.04	3.28
C17∶1	2.65	0.94	2.76
C18∶0	10.02	3.58	4.81
C18∶1n9t	1.54	0.55	2.69
C18∶1n9c	33.29	11.83	5.90
C18∶2n6t	0.47	0.17	2.30
C18∶2n6c	23.35	8.32	5.28
C20∶0	0.67	0.24	2.36
C18∶3n6	1.76	0.60	2.11
C20∶1	6.38	2.25	3.41
C18∶3n3	6.05	2.23	3.69
C21∶0	0.88	0.31	1.96
C20∶2	4.24	1.49	2.20
C22∶0	0.44	0.15	2.11
C20∶3n6	2.33	0.82	2.69
C22∶1n9	4.37	1.55	3.69
C20∶3n3	1.25	0.45	3.40
C20∶4n6	0.13	0.05	2.96
C23∶0	10.44	3.78	3.55
C22∶2	1.05	0.37	2.69
C24∶0	0.15	0.05	2.30
C20∶5n3 EPA	55.78	19.74	2.96
C24∶1	0.84	0.31	3.11
C22∶6n3 DHA	67.57	24.02	5.36
∑SFA	47.46	17.01	
∑MUFA	69.61	24.72	
∑PUFA	163.96	58.27	
ω-3/ω-6	4.74		
Total	281.03	100.00	

SFA: saturated fatty acids; MUFA: monounsaturated fatty acids; PUFA: polyunsaturated fatty acids; *ω*-3/*ω*-6: double bond position in PUFA.

## 3 结论

本研究建立了测定中华绒螯蟹肌肉中总脂肪酸组成的气相色谱分析方法。该方法测得中华绒螯蟹肌肉中31种脂肪酸,其中DHA、EPA等为中华绒螯蟹肌肉中主要脂肪酸。该方法操作简便,定性可靠,定量准确,能够反映中华绒螯蟹肌肉中丰富多样的脂肪酸组成与含量,具有一定的优越性。
